# AMPK Phosphorylation Impacts Apoptosis in Differentiating Myoblasts Isolated from Atrophied Rat Soleus Muscle

**DOI:** 10.3390/cells12060920

**Published:** 2023-03-16

**Authors:** Natalia A. Vilchinskaya, Sergey V. Rozhkov, Olga V. Turtikova, Timur M. Mirzoev, Boris S. Shenkman

**Affiliations:** Myology Laboratory, Institute of Biomedical Problems RAS, 123007 Moscow, Russia; rozhkov.work@yandex.ru (S.V.R.); olga_tur@list.ru (O.V.T.); tmirzoev@yandex.ru (T.M.M.); bshenkman@mail.ru (B.S.S.)

**Keywords:** AMPK, AICAR, primary myoblasts, hindlimb suspension, apoptosis, caspase-3, TUNEL

## Abstract

Regrowth of atrophied myofibers depends on muscle satellite cells (SCs) that exist outside the plasma membrane. Muscle atrophy appears to result in reduced number of SCs due to apoptosis. Given reduced AMP-activated protein kinase (AMPK) activity during differentiation of primary myoblasts derived from atrophic muscle, we hypothesized that there may be a potential link between AMPK and susceptibility of differentiating myoblasts to apoptosis. The aim of this study was to estimate the effect of AMPK activation (via AICAR treatment) on apoptosis in differentiating myoblasts derived from atrophied rat soleus muscle. Thirty rats were randomly assigned to the following two groups: control (C, n = 10) and 7-day hindlimb suspension (HS, n = 20). Myoblasts derived from the soleus muscles of HS rats were divided into two parts: AICAR-treated cells and non-treated cells. Apoptotic processes were evaluated by using TUNEL assay, RT-PCR and WB. In differentiating myoblasts derived from the atrophied soleus, there was a significant decrease (*p* < 0.05) in AMPK and ACC phosphorylation in parallel with increased number of apoptotic nuclei and a significant upregulation of pro-apoptotic markers (caspase-3, -9, BAX, p53) compared to the cells derived from control muscles. AICAR treatment of atrophic muscle-derived myoblasts during differentiation prevented reductions in AMPK and ACC phosphorylation as well as maintained the number of apoptotic nuclei and the expression of pro-apoptotic markers at the control levels. Thus, the maintenance of AMPK activity can suppress enhanced apoptosis in differentiating myoblasts derived from atrophied rat soleus muscle.

## 1. Introduction

Skeletal muscle satellite cells (SCs), also called muscle stem cells, are known to play a crucial role in muscle fiber maintenance, regeneration and (re)growth. Under unstressed conditions, SCs, located at the periphery of myofibers under the basal lamina, exist in a quiescent state (i.e., G_0_ phase of the cell cycle). However, upon stimulation, SCs exit their quiescent state and start to proliferate and differentiate. Differentiated myoblasts (the progeny of SCs) can fuse either into existing myofibers or with each other, forming new muscle fibers [1,2].

Inactivity/mechanical unloading is known to result in a decrease in the number of SCs in postural muscles [3,4,5,6]. Evidence suggests that SC depletion under degenerative conditions (Duchenne muscular dystrophy, chronic muscle denervation, and aging) can be related, at least partially, to apoptosis [7,8]. Available data concerning SC function under unloading/disuse conditions are contradictory. Some authors report a decrease in SC proliferation and differentiation under conditions of mechanical unloading [4,9], while others mark an increase in the activity of muscle SCs [10,11,12].

AMP-activated protein kinase (AMPK) is a well-known cell energy gauge, the activity of which is determined by the AMP:ATP ratio in the cell [13,14]. Under conditions of energy deprivation, AMPK is known to play an important role in the regulation of pathways related to fatty acid and cholesterol metabolism, mitochondrial biogenesis, anabolism, catabolism, autophagy, and coordination of cell survival [15]. In addition, the lack of AMPK in SCs can block normal muscle regeneration after injury [16]. It has been shown that in AMPKα1 knockout mice, skeletal muscle regeneration following injury is significantly weakened compared to wild-type mice. Moreover, AMPKα1 knockout SCs have reduced myogenic capacity when transplanted into wild-type muscles, suggesting that impaired muscle regeneration could be linked to the absence of AMPKα1 in the SCs [17]. Fu et al. (2015) have demonstrated that in response to muscle injury, AMPKα1 can serve as a critical mediator linking a non-canonical Sonic hedgehog pathway to Warburg-like glycolysis in SCs, thereby contributing to the activation of muscle stem cells skeletal muscle regeneration [18].

Thus, a certain level of AMPKα1 appears to be required for proper skeletal muscle regeneration following injury [19]. It has also been demonstrated that the loss of AMPK activity is a major reason for impaired muscle regeneration in obese mice [17]. However, hyperactivation of AMPK is able to impair SC proliferation and differentiation [20,21]. Furthermore, AMPK is involved in the regulation of apoptosis that normally accompanies myogenic differentiation of myoblasts [15]. Niesler et al. (2007) have shown that some level of AMPK activity is needed to inhibit apoptotic processes in differentiated C2C12 myotubes [22]. Moreover, we have recently found an accelerated differentiation and myotube formation in primary myoblasts derived from rat soleus muscles that were exposed to mechanical unloading prior [23]. We also observed decreased phosphorylation levels of acetyl-CoA carboxylase (ACC), a marker of AMPK activity, in rat soleus-derived myoblasts at later stages (5 days) of differentiation [24].

It is also known that denervation-induced muscle atrophy can increase susceptibility of SCs to apoptosis [8,25]. Since we recently found a decrease in AMPK activity during enhanced differentiation of primary myoblasts derived from atrophic rat soleus muscle [24], we hypothesized that there may be a potential link between AMPK activity and susceptibility of differentiating myoblasts to apoptosis. Hence, using AICAR, a specific AMPK activator, we aimed to estimate the effect of AMPK activation on apoptosis in differentiating myoblasts derived from atrophied rat soleus muscle.

## 2. Materials and Methods

### 2.1. Experimental Design

**Figure 1:**
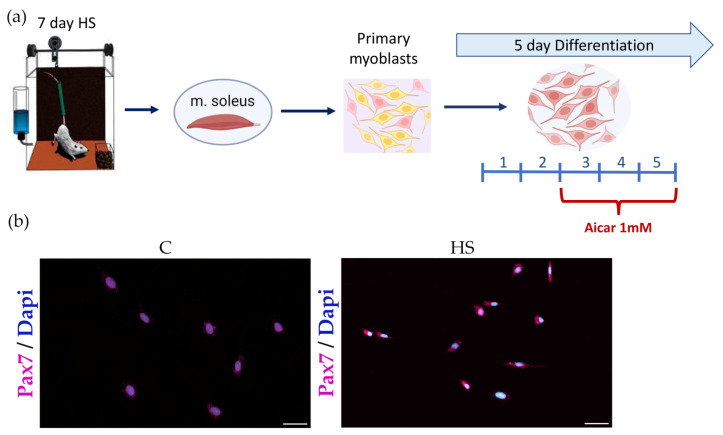
Experimental design. (**a**) Pax7 immunostaining in soleus-derived myoblasts (24 h in culture) and (**b**) Seven arbitrary fields were counted using a 20× objective. Approximately 93% of the cells are Pax7 positive. Pax7: violet, DAPI: blue. Scale bar = 50 μm.

A widely recognized Morey-Holton hindlimb suspension (HS) rodent model was used to induce mechanical unloading [26]. Male Wistar rats (3 months, 180 ± 10) were kept under standard laboratory conditions (room temperature about 21 °C and 12:12 h light/dark cycle) with free access to food and water. The rats were divided into three groups (n = 10/group): (1) control (C); (2) hindlimb suspension (HS); and (3) hindlimb suspension + AICAR (HS + AICAR). Upon completion of the HS experiment, soleus muscles from both hindlimbs were collected and subsequently used for isolation of muscle stem/progenitor cells. Euthanasia of animals was performed by decapitation under isoflurane anesthesia. Isolation of SCs/myoblasts from the soleus muscle was performed as described in our previous paper [23]. Following isolation, more than 90% of the isolated cells expressed Pax7 (Figure 1b). After obtaining the pure primary myoblast culture, the cells were cultured in growth medium under a humidified atmosphere with 5% CO_2_ at 37 °C. Two or three days later, when cells reached 80% confluency, myogenic differentiation was induced by changing the media to differentiation media (DMEM medium supplemented with 4.5 g/L D-glucose, L-glutamine, penicillin–streptomycin, and 2% of horse serum). Cells from the HS + AICAR group were incubated with differentiation media containing 1 mM AICAR (cat. ab120358, Abcam, Cambridge, UK) from day 3 to day 5 of differentiation (Figure 1a). All measurements in the study were performed on the 5th day of myogenic differentiation.

### 2.2. In Situ Detection of Apoptotic Cells

The detection of DNA double-strand breaks in differentiating myoblasts was performed using the terminal deoxynucleotidyl transferase dUTP nick-end labeling (TUNEL) technique, as described in Sancilio et al., (2022) [27]. Myotubes were fixed for 30 min in 4% paraformaldehyde at RT. They were then rinsed in PBS and incubated in a permeabilizing solution (0.1% Triton X-100 and 0.1% sodium citrate) for 2 min on ice. DNA strand breaks were determined using the In Situ Cell Death Detection Kit (cat. 11684795910, Roche, Basilea, Switzerland) according to the protocol supplied by the manufacturer. Nuclei were stained with 4′,6-diamidino-2-phenylindole (DAPI) (cat. D1306, Molecular Probes, Waltham, MA, USA). Fluoromount™ Aqueous Mounting Medium (cat. F4680, Sigma-Aldrich, Saint Louis, MA, USA) was used for mounting coverslips on slides. The Olympus inverted fluorescent microscope (20× magnification) and Cell Sens Dimension Software 3.2 (Build 23706) (Olympus, Tokyo, Japan) were used to acquire and analyse microscopic images.

### 2.3. Immunocytochemistry for Pax7 Detection

Detection of Pax7 in rat satellite cells was performed as described in our previous study [23].

### 2.4. Gene Expression Analysis

mRNA expression of target genes was determined by reverse transcription polymerase chain reaction (RT-PCR) as described in our previous study [23]. Primer sequences are provided in Table 1. Gapdh and Ywhaz were used as reference genes.

### 2.5. Western Blot Analysis

Western blot analysis was performed as described in our previous studies [28,29]. Primary antibodies used in the study were as follows: p-AMPK (Thr172) (1:500, cat. # Y408289, ABM, Richmond, BC, Canada), t-AMPK (1:1000, cat. # 2523, Cell Signaling Technology, Danvers, MA, USA), p-ACC (S79) (1:1000, cat. # 2535, Cell Signaling Technology, USA), t-ACC (1:1000, cat. # 3662, Cell Signaling Technology, USA), Caspase-3 (1:1000, cat. # 9661, Cell Signaling Technology, USA), p-rpS6 (S240/244) (1:2000, cat. # 5364 Cell Signaling Technology, Danvers, MA, USA), rpS6 (1:2000, cat. # 2217, Cell Signaling Technology, Danvers, MA, USA), Bax (1:2000, cat. # ab32503 Abcam, Cambridge, UK) and tubulin (1:3000, cat. # CSB-MA000185 Cusabio Biotech, Wuhan City, China). Horseradish peroxidase-conjugated antibodies to rabbit immunoglobulins (1:60000, cat. # 111-035-003, Jackson Immuno Research, Cambridge, UK) were used as secondary antibodies. Following image capture of phosphorylated proteins, membranes were stripped of the phospho-specific antibodies using RestoreTM Western Blot Stripping Buffer (cat. # 21059, Thermo Fisher Scientific, Waltham, MA, USA). The membranes were then re-probed with primary antibodies for each respective total protein. A total protein staining (Ponceau S) and/or tubulin protein expression were used for normalization of Western blots.

### 2.6. Statistical Analysis

Statistical analysis was performed using SigmaPlot 12.5 software. qRT-PCR and Western blot data are shown as mean ± SEM. Two-way ANOVA with post-hoc Tukey test was used to determine the significant differences between group means. Statistical significance was accepted at *p* < 0.05.

## 3. Results

### 3.1. Body Weight and Soleus Muscle Weight

Seven-day HS induced a slight decrease in rat body weight and a more profound reduction in absolute and normalized soleus muscle weight compared to the control group (Table 2).

### 3.2. Phosphorylation Status of AMPK (Thr172), ACC (Ser79) and rpS6 (Ser 240/244) in Differentiating Myoblasts

**Figure 2:**
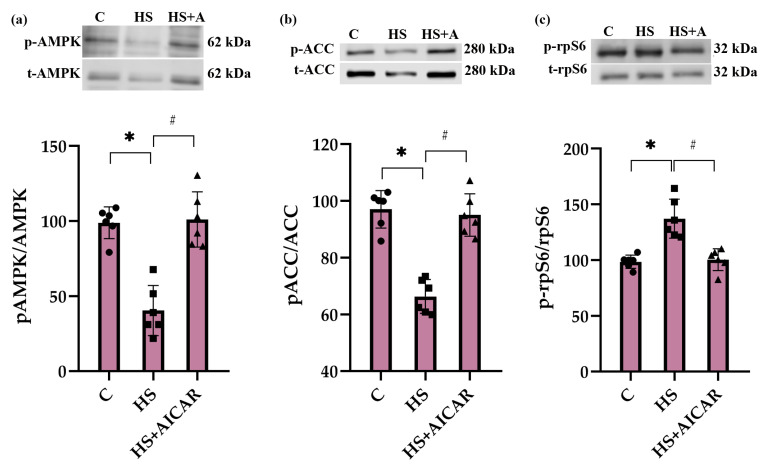
Effect of AICAR treatment on phosphorylation status of (**a**) AMPK (Thr172), (**b**) ACC (Ser79) and (**c**) rpS6 (Ser 240/244) in differentiating myoblasts derived from the atrophied rat soleus muscle. C—myoblasts derived from rat soleus muscle of the control rats, HS—myoblasts derived from rat soleus muscle after 7-day HS, HS+AICAR—myoblasts derived from rat soleus muscle after 7-day HS and treated with AICAR. Values are means ± SEM, expressed as % of the C. Dots, squares and triangles on the bars represent individual data points. n = 6, *—*p* < 0.05 vs. C; #— *p* < 0.05 vs. HS.

In the HS myoblasts, there was a significant decrease (53%) in the level of AMPK (Thr172) phosphorylation compared to the control myoblasts (Figure 2a). Treatment of the HS myoblasts with 5-Aminoimidazole-4-carboxamide ribonucleoside (AICAR) (a potent AMPK activator) prevented a decrease in AMPK (Thr172) phosphorylation (Figure 2a). Phosphorylation of ACC on Ser79 was reduced in the HS myoblasts and AICAR prevented this reduced ACC phosphorylation (Figure 2b). Furthermore, phosphorylation status of ribosomal protein S6 (rpS6), a marker of mTORC1 activity, was evaluated. In the HS myoblasts, there was a significant increase (29%) in rpS6 (Ser 240/244) phosphorylation compared to the control myoblast cultures. Treatment of the HS myoblasts with AICAR abrogated the increased rpS6 (Ser 240/244) phosphorylation (Figure 2c).

### 3.3. Effect of AICAR Treatment on Morphological Characteristics and Expression of Differentiation Markers in Myoblasts Derived from the Atrophied Rat Soleus Muscle

On the 5th day of differentiation, myotubes derived from the atrophied soleus muscle showed an increased fusion index but significantly decreased area, diameter, width and length relative to myotubes derived from the control soleus muscle (Table 3). AICAR treatment of differentiating myoblasts derived from the atrophied muscle reversed changes in fusion index and attenuated or fully prevented morphological alterations in myotubes (Table 3).

By the 5th day of myogenic differentiation, RT-PCR analysis also revealed that HS cells exhibit a significant upregulation of genes responsible for myoblast differentiation (myogenin and MyoD) and fusion (Myomaker and Myomixer) compared to the control cells (Table 4). However, treatment of differentiating HS myoblasts with AICAR significantly attenuated the expression of differentiation and fusion markers (Table 4).

### 3.4. The Number of Apoptotic Cells in Myoblast Cultures

**Figure 3:**
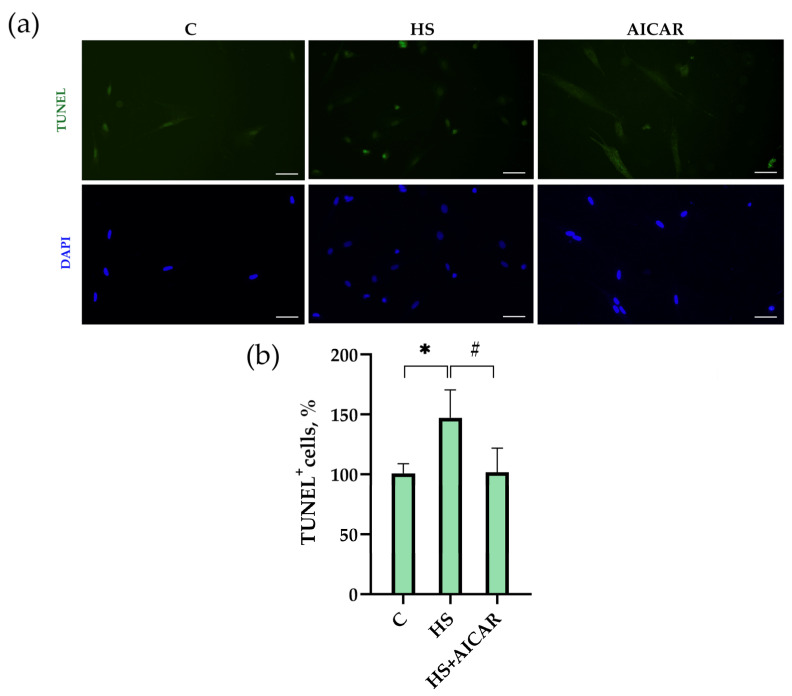
Effect of AICAR treatment on the number of apoptotic cells in differentiating myoblasts derived from the atrophied rat soleus muscle. (**a**) Representative images of TUNEL-stained (green labeling) and DAPI-stained (blue labeling) myoblasts are shown above the graph. (**b**) Quantification of TUNEL-positive cells. Scale bar= 50 μm. C—myoblasts derived from rat soleus muscle of the control rats, HS—myoblasts derived from rat soleus muscle after 7-day HS, HS+AICAR—myoblasts derived from rat soleus muscle after 7-day HS and treated with AICAR. Values are means ± SEM, expressed as % of the C. *—*p* < 0.05 vs. C; #— *p* < 0.05 vs. HS.

TUNEL assay revealed that in the HS myoblasts, the number of apoptotic cells was 43% greater than in the control myoblasts (Figure 3). The number of apoptotic cells in the AICAR-treated HS myoblasts did not differ from the control myoblasts (Figure 3). Thus, the maintenance of AMPK activity in differentiating myoblasts derived from the atrophied soleus muscle was able to fully prevent the increased number of TUNEL^+^ cells.

### 3.5. Expression of Apoptotic Markers in Myoblast Cultures

**Figure 4:**
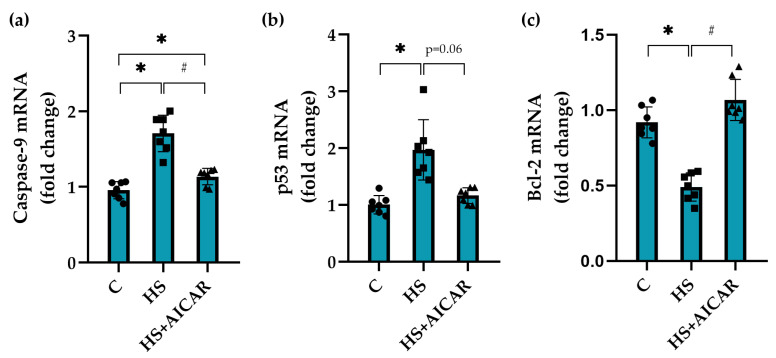
Effect of AICAR treatment on the mRNA expression of (**a**) pro-apoptotic markers (*caspase-9*, (**b**) *p53* and (**c**) anti-apoptotic marker (*Bcl-2*) in differentiating myoblasts derived from the atrophied rat soleus muscle. C—myoblasts derived from rat soleus muscle of the control rats, HS—myoblasts derived from rat soleus muscle after 7-day HS, HS+AICAR—myoblasts derived from rat soleus muscle after 7-day HS and treated with AICAR. Values are means ± SEM, expressed as fold changes vs. C. Dots, squares and triangles on the bars represent individual data points. n = 7, *—*p* < 0.05 vs. C; # – *p* < 0.05 vs. HS.

In the HS myoblasts, there was an increase in *caspase-9* mRNA expression by 78% (Figure 4a) and *p53* mRNA expression by 100% (Figure 4b) compared to the C myoblasts. In the AICAR-treated HS myoblasts, no significant changes in the expression levels of *caspase-9* and *p53* were found relative to the C myoblasts (Figure 4a,b). In the HS myoblasts, mRNA expression levels of *Bcl-2*, a protein that inhibits apoptosis, were reduced by 45% compared to the control myoblasts (Figure 4c). The expression levels of *Bcl-2* after AICAR treatment of the HS myoblasts did not differ from the control myoblasts (Figure 4c).

**Figure 5:**
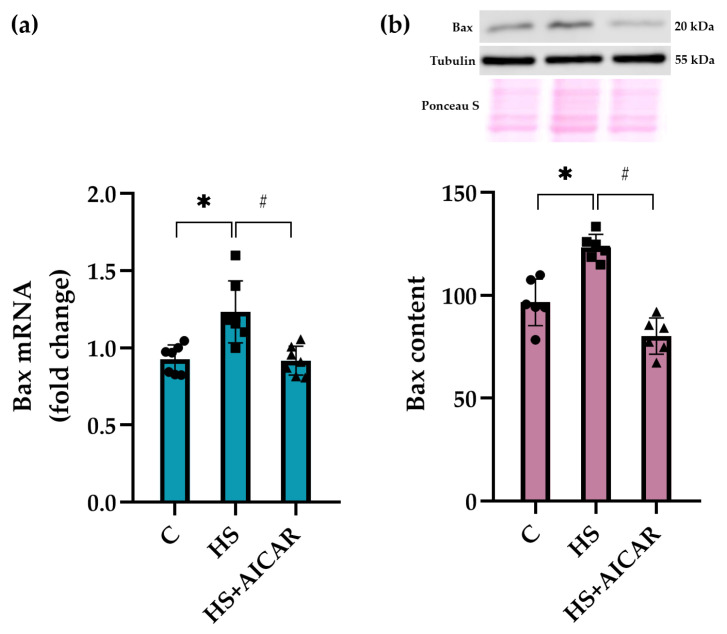
Effect of AICAR treatment on (**a**) *Bax* mRNA expression and (**b**) Bax protein content in differentiating myoblasts derived from the atrophied rat soleus muscle. C—myoblasts derived from rat soleus muscle of the control rats, HS—myoblasts derived from rat soleus muscle after 7-day HS, HS+AICAR—myoblasts derived from rat soleus muscle after 7-day HS and treated with AICAR. Values are means ± SEM, expressed as fold changes vs. C (PCR data, n = 7) or as % of C (WB data, n = 6). Dots, squares and triangles on the bars represent individual data points. *—*p* < 0.05 vs. C; #– *p* < 0.05 vs. HS.

In the HS cells, we also observed a significant upregulation of pro-apoptotic marker Bax at both mRNA and protein expression levels compared to the control cells (Figure 5). AICAR treatment of HS myoblasts fully prevented this increased *Bax* mRNA expression and protein abundance (Figure 5). Thus, differentiating myoblasts isolated from the unloaded soleus muscle showed a significant increase in the expression of pro-apoptotic markers (*caspase-9, p53,* and *Bax*) and a concomitant decrease in the expression of anti-apoptotic marker (*Bcl-2*). AICAR-induced prevention of AMPK dephosphorylation in the HS myoblasts resulted in the maintenance of the apoptotic markers’ expression at the control levels.

**Figure 6:**
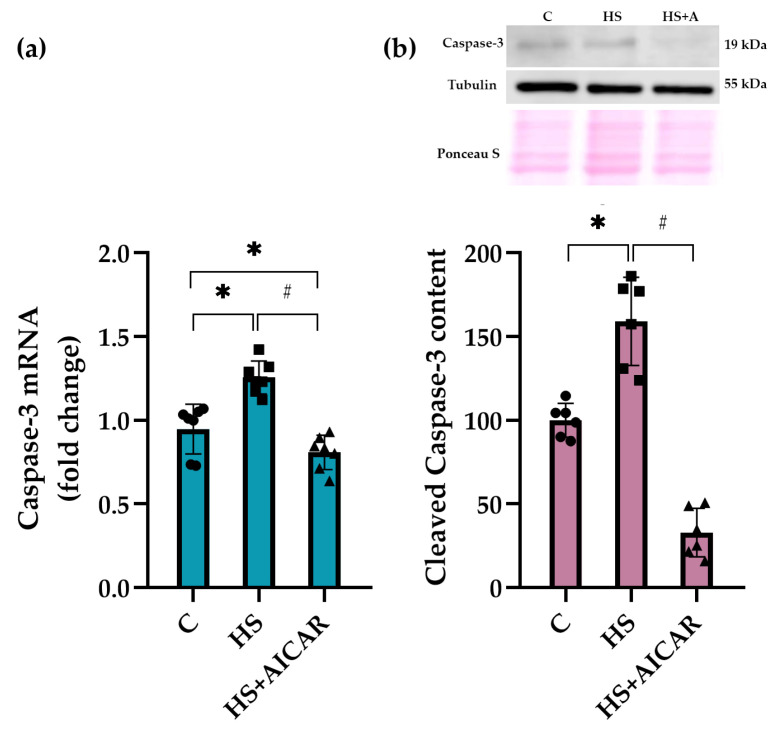
Effect of AICAR treatment on (**a**) *caspase-3* mRNA expression and (**b**) cleaved caspase-3 protein content in differentiating myoblasts derived from the atrophied rat soleus muscle. C—myoblasts derived from rat soleus muscle of the control rats, HS—myoblasts derived from rat soleus muscle after 7-day HS, HS+AICAR—myoblasts derived from rat soleus muscle after 7-day HS and treated with AICAR. Values are means ± SEM, expressed as fold changes vs. C (PCR data, n = 7) or as % of C (WB data, n = 6). Dots, squares and triangles on the bars represent individual data points. *—*p* < 0.05 vs. C; # – *p* < 0.05 vs. HS.

We also evaluated both mRNA expression levels and the content of cleaved caspase-3, a key effector enzyme in apoptosis induction, in differentiating myoblast cultures. As shown in Figure 4a, mRNA expression levels of *caspase-3* were significantly upregulated in the HS myoblasts compared to the C cultures. However, AICAR treatment lowered *caspase-3* mRNA expression levels below the C values (Figure 6a). The content of cleaved caspase-3 in the HS myoblasts was 53% greater relative to the C myoblasts (Figure 6b). However, in the HS+AICAR myoblasts, the content of cleaved caspase-3 was significantly lower than in the C and HS myoblast cultures (Figure 6b). These data support the findings presented in Figure 3, Figure 4 and Figure 5 about the activation of apoptotic processes in differentiating myoblasts derived from the atrophied rat soleus muscle.

## 4. Discussion

Our data demonstrate, for the first time, the direct effects of AICAR treatment (and, hence, the maintenance of AMPK activity) on the apoptosis in differentiating primary myoblasts derived from mechanically unloaded/atrophied rat soleus muscle. Previous studies demonstrated that exposure to mechanical unloading/microgravity can lead to apoptotic processes in skeletal muscles [30]. Radugina et al. (2017) have shown that exposure of mice to 30-day microgravity results in the presence of multiple apoptotic nuclei and a smaller number of SCs in quadriceps muscles compared to the control mice [10]. It is known that a certain level of apoptosis normally accompanies myoblast differentiation [31,32]. Enhanced apoptosis during myoblast differentiation can contribute to muscle fiber degeneration, as was revealed in various types of muscular dystrophy and atrophy cases [33,34,35]. In the present study, we found a significant upregulation of apoptosis during differentiation of primary myoblasts isolated from rat soleus after 7-day mechanical unloading. Using TUNEL assay, we identified a greater number of apoptotic cells in the culture of differentiating myoblasts derived from the atrophic soleus muscles compared to the differentiating myoblasts derived from the control soleus muscles. Moreover, the levels of mRNA expression of pro-apoptotic markers (*caspase-3* and *-9, p53* and *Bax*) were significantly increased in parallel with reduced mRNA expression of anti-apoptotic *Bcl-2*. The presence of apoptosis in these HS myoblasts was also confirmed by an increased content of cleaved caspase-3. These results correlate well with some literature data. For instance, Andrianjafiniony et al. (2010) have showed a significant increase in the content of caspase-3 and -9 in rat soleus muscle after 14 days of HS [36], which is in agreement with the above mentioned study by Radugina (2017) on the effect of 30-day unloading (microgravity) on murine skeletal muscle [10]. Furthermore, a significant increase in apoptosis was shown in differentiated myotubes derived from skeletal muscles of patients with myotonic dystrophy [37].

We also determined the levels of AMPK activity (by assessing AMPK Thr172 phosphorylation and ACC Ser 79 phosphorylation) since it is known that AMPK can contribute to the regulation of programmed cell death (apoptosis) normally, accompanying myogenesis and muscle regeneration [15,22,38]. Available data on the role of AMPK in the regulation of apoptosis are controversial. While some reports suggest AMPK-dependent stimulation of apoptosis [39,40,41], several studies demonstrate an anti-apoptotic role of AMPK [22,42,43,44].

In the present study, we found a significant decrease in both AMPK (Thr 172) phosphorylation and ACC (Ser 79) phosphorylation in differentiating myoblasts derived from rat soleus muscle after 7-day HS, indicative of a reduction in AMPK kinase activity. Moreover, a decrease in the activity of AMPK signaling was confirmed by a significant increase in Ser 240/244 phosphorylation of rpS6, a marker of mTORC1 activity, since AMPK is known to be an endogenous inhibitor of mTORC1 and protein synthesis in both skeletal muscles [45] and cultured muscle cells [46,47]. These data are consistent with our previous study showing a decrease in ACC (Ser 79) phosphorylation in primary myoblasts isolated from unloaded skeletal muscle [24]. The results on the activity of AMPK and ACC in differentiating myoblasts derived from soleus muscle after mechanical unloading are in agreement with data previously obtained directly on rat soleus muscle [28,29,48]. However, these unloading-induced changes in AMPK activity were seen in rat soleus muscle at earlier stages of HS (1–3 days) compared to differentiating myoblasts isolated from rat soleus muscle after 7-day HS in the present study. Liu et al. (2019) have previously demonstrated, although in non-muscle cells, that AMPK knockdown results in the upregulation of apoptosis [49]. In primary myoblast derived from geriatric skeletal muscle, White and colleagues (2018) have demonstrated that reduced AMPK activity (phosphorylation) is associated with increased apoptosis [44]. Moreover, it has also been shown in differentiating C2C12 myoblasts that an inhibition of AMPK activity contributes to apoptosis upregulation [22].

In the present study, to elucidate the role of AMPK activity in the regulation of apoptosis in differentiating myoblasts derived from the unloaded/atrophied soleus muscle, a specific AMPK activator, AICAR, was used. Incubation of the HS myoblasts with AICAR not only prevented HS-induced reductions in the phosphorylation levels of AMPK and ACC, but also reduced the number of apoptotic cells and maintained the expression of apoptotic markers at the control levels. Thus, our data clearly demonstrate that the maintenance of AMPK activity (Thr 172 phosphorylation) prevents apoptosis development in differentiating myoblasts derived from the rat soleus after 7-day mechanical unloading.

It is known that AMPK can participate in the regulation of apoptosis through different mechanisms, including regulation of autophagy [50], Bcl-2-regulated apoptotic pathway [51], mTOR inhibition [52], p53 activation [53] and phosphorylation of cyclin-dependent kinase inhibitor 1B (p27Kip1) [44]. Under conditions of metabolic stress, p27Kip1 is involved in the regulation of cell fate. In particular, p27Kip1 controls cell cycle inhibition, apoptosis and autophagy [38]. It has been shown that p27Kip1 can inhibit the activity of pro-apoptotic protein Bax and prevent apoptosis [54,55]. The regulation of p27Kip1 activity is carried out at the level of transcription, phosphorylation and subcellular localization [44]. It has been demonstrated that nuclear p27Kip1 is able to facilitate quiescence and apoptosis, while cytoplasmic p27Kip1 can promote cell survival and autophagy [44]. Liang and co-workers demonstrated that AMPK-related phosphorylation of p27Kip1 on Thr198 is able to stimulate its sequestration to the cytosol, leading to increased autophagy and decreased apoptosis [38]. In myoblasts derived from aged mice, increased apoptosis was accompanied by decreased AMPK and p27Kip1 phosphorylation [44]. Moreover, upon AMPK activation or p27Kip1 overexpression, apoptosis in these cells was suppressed [44]. We can speculate that in the present study, the development of apoptosis in differentiating myoblasts derived from the unloaded soleus muscle could occur due to reduced AMPK-dependent p27Kip1 phosphorylation, resulting in p27Kip1 nuclear localization and induction of apoptosis. Identification of the precise molecular mechanisms underlying apoptosis development in differentiating myoblasts isolated from atrophied muscles requires further research.

In conclusion, our study for the first time revealed the increase in apoptotic processes in differentiating myoblasts derived from the atrophied rat soleus muscle. Further, our data provide the first evidence that the activation of apoptosis in such differentiating myoblasts is associated, at least in part, with reduced AMPK activity.

## Figures and Tables

**Table 1:** Primer sequences for RT-PCR analysis.

Gene Name	Sequence (5’->3’)	GenBank
*Caspase 9*	5’-gaagaacgacctgactgctaag-3’ 5’-atgagagaggatgaccacca-3’	NM_031632.2
*Caspase 3*	5’-gagcttggaacgcgaagaaa-3’ 5’-taaccgggtgcggtagagta-3’	NM_012922.2
*p53*	5’-cccctgaagactggataactgt-3’ 5’-gacctcaggtggctcatacg-3’	NM_030989.3
*Bax*	5’-ggcctttttgctacagggtttc-3’ 5’-gggggtcccgaagtaggaaag-3’	NM_017059.2
*Bcl-2*	5’-tcatgtgtgtggagagcgtc-3’ 5’-agttccacaaaggcatcccag-3’	NM_016993.2
*Ywhaz*	5’-cccactccggacacagaata-3’ 5’-tgtcatcgtatcgctctgcc-3’	NM_013011.4
*Gapdh*	5′-cggtgtgaacggatttggc-3′ 5′-ttgaggtcaatgaaggggtcg-3′	NM_017008.4

**Table 2:** Changes in body weight, soleus wet weight and soleus weight-to-body weight ratio. C—control group, HS—hindlimb suspension for 7 days. Values are means ± SEM. * —*p* < 0.05 vs. C.

Groups	Body Weight, g	Soleus Wet Weight, mg	Soleus Weight-to-Body Weight Ratio, mg/g
C	235 ± 4	78 ± 3	0.33 ± 0.01
HS	213 ± 5 *	49 ± 3 *	0.23 ± 0.01 *

**Table 3:** Morphological characteristics of differentiating myoblasts derived from the atrophied rat soleus muscle. Values are means ± SEM. The fusion index was calculated as the percentage of nuclei in fused myotubes out of the total nuclei, measured in %. C—myoblasts derived from rat soleus muscle of the control rats, HS—myoblasts derived from rat soleus muscle after 7-day HS, HS+AICAR—myoblasts derived from rat soleus muscle after 7-day HS and treated with AICAR; n ≥ 36/group. *—significant difference vs. C, *p* < 0.05, #—significant difference vs. HS, *p* < 0.05.

Groups	Fusion Index, %	Area, µm²	Diameter, µm	Width, µm	Length, µm
C	100 ± 4.3	8908 ± 814	70 ± 3	25 ± 1	510 ± 30
HS	143 ± 13.5 *	5667 ± 569 *	54 ± 3 *	17 ± 1 *	306 ± 38 *
HS+ AICAR	102 ± 10.2 ^#^	7686 ± 759 ^#^	69 ± 3 ^#^	24 ± 2 ^#^	365 ± 31 *

**Table 4:** Expression level of differentiation markers in myoblasts derived from the atrophied rat soleus muscle. Values are means ± SEM. C—myoblasts derived from rat soleus muscle of the control rats, HS—myoblasts derived from rat soleus muscle after 7-day HS, HS+AICAR—myoblasts derived from rat soleus muscle after 7-day HS and treated with AICAR; n = 7/group. *—significant difference vs. C, *p* < 0.05, #—significant difference vs. HS, *p* < 0.05.

Groups	Myogenin mRNA, 2^−ΔΔCt^	MyoD mRNA, 2^−ΔΔCt^	Myomaker mRNA, 2^−ΔΔCt^	Myomixer mRNA, 2^−ΔΔCt^
C	1 ± 0.06	1 ± 0.04	1 ± 0.02	1 ± 0.05
HS	2.91 ± 0.22 *	2.49 ± 0.28 *	1.4 ± 0.15 *	2.02 ± 0.11 *
HS + AICAR	1.78 ± 0.15 *^#^	1.63 ± 0.27 ^#^	1.14 ± 0.06 ^#^	1.67 ± 0.19 *

## Data Availability

The data presented in the study are available upon reasonable request from the corresponding author.
